# An Anti-Inflammatory Diet and Its Potential Benefit for Individuals with Mental Disorders and Neurodegenerative Diseases—A Narrative Review

**DOI:** 10.3390/nu16162646

**Published:** 2024-08-10

**Authors:** Sophie M. van Zonneveld, Ellen J. van den Oever, Benno C. M. Haarman, Emmy L. Grandjean, Jasper O. Nuninga, Ondine van de Rest, Iris E. C. Sommer

**Affiliations:** 1 Department of Biomedical Sciences, Cognitive Neuroscience Center, University Medical Center Groningen, University of Groningen, 9713 GZ Groningen, The Netherlands; 2 Department of Psychiatry, University Medical Center Groningen, University of Groningen, 9713 GZ Groningen, The Netherlands; 3 Department of Human Nutrition and Health, Wageningen University & Research, 6708 WE Wageningen, The Netherlands

**Keywords:** mental disorders, neurodegenerative diseases, inflammation, gut microbiome, anti-inflammatory diet, food groups

## Abstract

This narrative review synthesizes current evidence regarding anti-inflammatory dietary patterns and their potential benefits for individuals with mental disorders and neurodegenerative diseases. Chronic low-grade inflammation is increasingly recognized as a key factor in the etiology and progression of these conditions. The review examines the evidence for the anti-inflammatory and neuroprotective properties of dietary components and food groups, focusing on whole foods rather than specific nutrients or supplements. Key dietary components showing potential benefits include fruits and vegetables (especially berries and leafy greens), whole grains, legumes, fatty fish rich in omega-3, nuts (particularly walnuts), olive oil, and fermented foods. These foods are generally rich in antioxidants, dietary fiber, and bioactive compounds that may help modulate inflammation, support gut health, and promote neuroprotection. Conversely, ultra-processed foods, red meat, and sugary beverages may be harmful. Based on this evidence, we designed the Brain Anti-Inflammatory Nutrition (BrAIN) diet. The mechanisms of this diet include the modulation of the gut microbiota and the gut–brain axis, the regulation of inflammatory pathways, a reduction in oxidative stress, and the promotion of neuroplasticity. The BrAIN diet shows promise as an aid to manage mental and neurodegenerative disorders.

## 1. Introduction

Nutrition plays a crucial role in maintaining health and well-being, with growing evidence suggesting its importance for brain function [1]. There has been increasing interest in the role of diet in managing and preventing various neurological and psychiatric disorders, particularly through anti-inflammatory effects [2]. Chronic low-grade inflammation is increasingly recognized as a factor in the etiology and progression of several mental disorders, including anxiety disorders, mood disorders, schizophrenia spectrum disorders (SSD), and neurodegenerative diseases such as Parkinson’s disease (PD) and Alzheimer’s disease (AD) [3,4,5,6].

Recent studies have highlighted the role of the gut–brain axis, emphasizing the bidirectional communication between the central nervous system and the gastrointestinal tract [7]. This axis involves neural, endocrine, and immune pathways [8]. Microorganisms residing in the digestive tracts are known as the gut microbiota, comprising predominantly bacteria, but also viruses, archaea, fungi and protozoa. The gut microbiota plays a vital role in the gut–brain axis by (a) aiding digestion by helping gut cells absorb nutrients and fermenting certain food components to produce metabolites such as short-chain fatty acids (SCFA); (b) aiding in the maturation of the digestive tract by helping form gastrointestinal mucus and enhancing mucosal enzyme activity; (c) serving as a barrier against pathogens and toxins by producing antimicrobial agents; (d) supporting immune system development; and (e) assisting in the synthesis of essential vitamins, such as vitamin B [9].

Dysbiosis, an imbalance in the composition and metabolism of the gut microbiota, may cause gastrointestinal disorders and impact other organs and systems [9]. Dysbiosis can lead to gut barrier dysfunction and endotoxemia, triggering low-grade systemic inflammation and neuroinflammation [10]. Such disruptions have been linked to increased blood–brain barrier permeability, mental disorders and neurodegenerative diseases [11,12,13,14,15]. Conversely, patients with these neurological conditions often experience gastrointestinal problems, like abdominal pain, bloating, constipation or diarrhea, which can further impair health [16,17]. A recent meta-analysis showed that depression was significantly associated with Crohn’s disease and ulcerative colitis, both types of inflammatory bowel disease (IBD) [18]. Gastrointestinal issues can impact medication absorption, nutritional status, employability and quality of life, highlighting the importance of dietary factors in the management of mental disorders and neurodegenerative diseases.

Diet significantly impacts the composition of the gut microbiota, with distinct differences observed between plant-based and animal-based diets [19]. Other factors influencing the gut microbiota include stress, environmental conditions, medications, life stages, and medical conditions. As gut bacteria need nutritional fibers (“prebiotics”) for their survival, a diet rich in different sources of fibers, stemming from whole grain, fruit and vegetables, benefits a diverse gut microbiome [20].

A diet supporting the immune system must contain few pro-inflammatory ingredients and many anti-inflammatory ingredients [21]. Food components can directly influence the immune system by activating or deactivating gut-dwelling immune cells through several receptors, one of them being the Toll-like receptors [22]. An activated immune system, often referred to as “inflammaging”, can cause tissue damage and increase the risk of cardiometabolic disorders and depression [23]. Pro-inflammatory foods typically include those high in saturated fats, trans fats, and refined sugars, such as processed meats, sugary beverages, and refined grains. Diets high in fat and sugar can result in gut dysbiosis, systemic inflammation, and neurodegeneration [24]. Anti-inflammatory foods include those rich in omega-3 fatty acids, antioxidants, and dietary fibers, such as fatty fish, leafy green vegetables, berries, nuts, and olive oil [25].

Certain dietary patterns and specific foods are associated with either increased or decreased levels of inflammatory markers. For instance, the Mediterranean diet, characterized by the high consumption of fruits, vegetables, whole grains, and olive oil, has been linked to lower levels of inflammatory markers and a reduced risk of various chronic diseases [26]. Conversely, diets high in processed foods, refined carbohydrates, and saturated fats have been associated with increased inflammation and a higher risk of neurological and psychiatric disorders [27].

The present review aims to synthesize evidence regarding the potential benefits of an anti-inflammatory diet for adults with mental or brain disorders. We will examine the scientific basis for the anti-inflammatory and neuroprotective properties of specific dietary components and food groups. The review focuses on whole foods and food groups rather than on specific nutrients or dietary supplements. We will additionally explore the mechanisms through which these dietary factors may influence brain health, including effects on the gut microbiota, the modulation of inflammatory pathways, and direct neuroprotective actions. After consolidating the available evidence, we propose the Brain Anti-Inflammatory Nutrition (BrAIN) diet, aimed at optimizing brain health for adults.

## 2. Evidence for Dietary Effects in Mental Disorders and Neurodegenerative Diseases

### 2.1. Mental Disorders

Emerging evidence suggests that dietary factors play a role in the risk of mental disorders and symptom management. A cross-sectional study found that a higher quality diet was associated with a reduced likelihood of bipolar disorder (BD) [28].

Epidemiological studies have suggested a link between dietary patterns and the risk or severity of SSD. A systematic review found that adherence to a healthy diet with a high intake of fruits, vegetables, and whole grains is associated with a reduced risk of psychosis and improved outcomes [29]. Conversely, a diet high in processed foods and saturated fats is associated with increased risk and worse outcomes.

Intervention studies targeting diet in BD are scarce. A small pilot study investigated the effects of a Mediterranean-style diet in 20 individuals with BD [30]. After 3 months, participants showed improvements in depressive symptoms and overall functioning.

Intervention studies in SSD are few but promising. A dietary intervention based on the Mediterranean diet [31] improved the cognitive function of patients with schizophrenia and metabolic syndrome after three months of dietary intervention, whereas those without the intervention did not see a change. A 4-month pilot study was conducted on 23 individuals with BD or schizophrenia and existing metabolic abnormalities [32]. A ketogenic diet improved metabolic health and resulted in a 32.6% improvement in the symptoms of schizophrenia [32]. However, the study was small and lacked a control arm.

### 2.2. Neurodegenerative Diseases

Dietary factors have been implicated in both the risk and progression of PD. A meta-analysis showed that a diet high in antioxidants reduced the risk of PD [33]. Several prospective cohort studies suggested that a higher intake of fruits, vegetables, and fish protect against PD [34,35].

Recent studies highlighted the benefits of the ‘MIND’ diet in reducing the risk of AD [36]. This diet blends elements of the Mediterranean and the Dietary Approaches to Stop Hypertension (DASH) diet. The MIND diet emphasizes green leafy vegetables, nuts, berries, beans, whole grains, fish, poultry, olive oil, and moderate wine consumption, while limiting red meats, butter, cheese, sweets, and fried foods [37]. An observational study found that high adherence to the MIND diet was linked to reduced cognitive decline [37]. High adherence to the MIND diet was linked to a 53% reduced risk of AD, and even moderate adherence offered a 35% risk reduction [37]. The diet was also associated with fewer Alzheimer’s-related brain plaques and tangles [37]. The MIND diet has anti-inflammatory properties and reduces oxidative stress, providing protection against AD [38]. Conversely, the Western diet, high in processed foods, can impair metabolic health and cerebral perfusion, increasing the risk of AD [38].

We found few intervention studies examining the effects of dietary changes on PD progression. A two-week dietary intervention study using anti-inflammatory food products in 16 patients with PD showed that dietary intervention is a sufficient method to improve the gut microbiome and reduces medication use [39]. Although the small sample of 16 patients with PD and the short duration of the intervention are important limitations of this study, the results are hopeful.

A recent RCT (n = 51) investigated the impact of intensive lifestyle changes, including a Mediterranean-style diet, on patients with mild cognitive impairment or early-stage AD [40]. The study found that those who adopted this healthy lifestyle showed improved cognitive function compared to those who did not. The Mediterranean diet was highlighted for its anti-inflammatory properties and benefits to brain health.

While these findings are encouraging, it is important to note that the evidence base for dietary interventions for treating mental disorders and neurodegenerative diseases is still developing. Most studies were observational in nature or evaluate small-scale interventions. Larger, well-designed RCTs are needed to establish causal relationships and determine the effective dietary approaches.

## 3. Potential Mechanisms of Anti-Inflammatory Diets

The benefits of an anti-inflammatory diet on mental disorders and neurodegenerative diseases are mediated through several interconnected mechanisms. Understanding these mechanisms can provide insight into how dietary interventions may influence disease progression and symptomatology.

A primary mechanism involves the modulation of the gut microbiota. The human gut hosts trillions of microorganisms that play crucial roles in digestion, immune function, and even neurotransmitter production [8]. Dietary components, particularly polyphenols and dietary fibers, can significantly influence the composition and function of this microbial community. For instance, a diet rich in diverse plant foods has been associated with increased microbial diversity, which is generally considered beneficial for health [41]. Isoflavones, belonging to the flavonoid family of polyphenols, act on the estrogen receptor and are known to be useful in the prevention and cure of noncommunicable diseases, including cardiovascular diseases (CVDs), metabolic syndrome and neurodegenerative diseases, particularly in post-menopausal women [42,43]. The gut microbiota’s influence extends beyond the gastrointestinal tract through the gut–brain axis. One key aspect of this axis is the production of SCFAs by gut bacteria through the fermentation of dietary fibers. SCFAs, particularly butyrate, have anti-inflammatory properties and may influence brain function and behavior [44]. Although direct clinical evidence is scarce, the dietary effects include improvements in symptoms such as fatigue, low or unstable mood, sensitivity to stress and cognitive dysfunctions.

A second critical mechanism is the modulation of inflammation pathways. Chronic low-grade inflammation including neuroinflammation is a common feature in many mental disorders and neurodegenerative diseases. Dietary components can either exacerbate or mitigate this inflammation. Anti-inflammatory diets rich in fruits, vegetables, whole grains, and omega-3 fatty acids have been associated with reduced levels of inflammatory markers including C-reactive protein (CRP), interleukin-6 (IL-6), and tumor necrosis factor-alpha (TNF-α) [26]. These effects may be particularly relevant in neuropsychiatric disorders, where neuroinflammation is increasingly recognized as a key pathological feature.

A third important mechanism is the regulation of oxidative stress, which is an imbalance between free radicals and antioxidants. Antioxidants are compounds that help neutralize harmful free radicals in the body, thereby preventing cellular damage. They can be classified into two main types: exogenous and endogenous antioxidants. Exogenous antioxidants, such as vitamins, minerals, polyphenols, cannot be synthesized by the body in sufficient quantities or at all, and must be ingested through diet. Endogenous antioxidants, such as superoxide dismutase (SOD), glutathione and ubiquinone, are produced naturally within the body [45]. Many components of an anti-inflammatory diet—including vitamins C and E, carotenoids, and polyphenols—act as antioxidants, helping to neutralize harmful free radicals directly [21] or, even more likely, via the elevation of the endogenous antioxidant defense induced by, for example, cruciferous vegetables including broccoli, cauliflower, cabbage, kale, brussels sprouts [45]. Oxidative stress has been implicated in the pathogenesis of many mental disorders and neurodegenerative diseases, with evidence of increased oxidative damage in brain tissues [46,47]. By reducing oxidative stress, these dietary components may help protect against neuronal damage and dysfunction.

Dietary factors may also influence neurotransmitter systems, which are often dysregulated in mental disorders and neurodegenerative diseases [48,49,50]. For example, tryptophan, an essential amino acid found in many protein-rich foods, is a precursor to serotonin, a neurotransmitter involved in mood regulation. Similarly, tyrosine, found for example in almonds and avocados, is a precursor to dopamine, which is particularly relevant in PD [51]. Moreover, the gut microbiota can influence neurotransmitter production and metabolism, providing another link between diet, the gut, and brain function.

Neuroprotection and neuroplasticity are additional mechanisms by which diet may influence these disorders. Certain dietary components, such as omega-3 fatty acids and flavonoids (polyphenolic compounds produced by plants), have been shown to promote the expression of neurotrophic factors like brain-derived neurotrophic factor (BDNF), which is crucial for neuronal survival and plasticity [52]. This protective effect may be particularly relevant in neurodegenerative conditions like PD, in which preserving neuronal function is a key therapeutic goal.

Finally, epigenetic modifications represent another mechanism linking diet to brain health. Dietary factors can influence gene expression through DNA methylation and histone modifications. These changes can affect the expression of genes involved in inflammatory responses, neurotransmitter function, and neuroprotection [53].

These mechanisms are not mutually exclusive but interact in complex ways. Moreover, individual variability in genetic makeup, gut microbiota composition, and environmental factors may influence how dietary interventions affect a person. This complexity underscores the need for personalized approaches to dietary interventions in people with a mental disorder or neurodegenerative disease. While our understanding of these mechanisms is growing, much remains to be elucidated. Future research using advanced techniques such as metabolomics, proteomics, and advanced neuroimaging will be crucial in further unraveling the complex relationships between diet, inflammation, and brain health in these disorders.

## 4. Key Food Groups and Components

Several food groups and dietary components have shown potential benefits for individuals with mental disorders and neurodegenerative diseases, primarily through their anti-inflammatory and neuroprotective properties, but also by providing a source of nutrition to gut bacteria and by providing important building blocks to sustain brain plasticity. Here, we outline key food groups and components, their effects, and the evidence supporting their inclusion in an anti-inflammatory diet for brain health.

### 4.1. Fruits and Vegetables

Fruits and vegetables are cornerstone components of anti-inflammatory diets. Rich in vitamins, minerals, dietary fibers, and bioactive compounds such as polyphenols, these foods have been associated with reduced risk and improved outcomes in several mental disorders and neurodegenerative diseases [54,55]. Their beneficial effects are likely mediated through multiple mechanisms. Firstly, antioxidants like vitamins C and E, carotenoids, and flavonoids help combat oxidative stress, a common feature of these disorders. Secondly, the nutritional fiber content supports gut health by promoting gut bacteria, producing beneficial microbial metabolites such as SCFAs, and potentially influencing the gut–brain axis [56]. Thirdly, many plant compounds exhibit direct anti-inflammatory actions, which may help mitigate chronic low-grade inflammation.

Among fruits, berries [57,58,59,60,61,62] are considered to have particularly strong anti-inflammatory and therefore neuroprotective effects. Red and blue fruits are rich in antioxidants and have been associated with improved cognitive function in several studies [37,63]. The consumption of green leafy vegetables, to some extent cruciferous vegetables, and of red/yellow vegetables is associated with slower cognitive decline rates [47,64,65,66,67].

In summary, the incorporation of a variety of fruits and vegetables, particularly those rich in antioxidants and dietary fibers, into one’s diet plays a pivotal role in maintaining or enhancing mental and brain health. These foods not only provide essential nutrients that support overall health, but also offer specific benefits through their anti-inflammatory and neuroprotective properties.

### 4.2. Whole Grains

Whole grains provide a complex array of nutrients, including dietary fiber, B vitamins, and various phytochemicals. These components may support gut health, reduce inflammation, and provide neuroprotective effects. While specific studies in patients with mental disorders and neurodegenerative diseases are limited in number or scope, wholegrain consumption has been associated with lower levels of inflammatory markers in general populations [68]. The dietary fiber content in wholegrains can also support a healthy gut microbiome, which is increasingly recognized as a factor in brain health. Dietary fibers promote intestinal health by modifying the gut microbial profile, producing SCFAs, increasing bowel movement frequency and relieving constipation, in addition to their anti-inflammatory properties [69,70,71,72]. Wholegrains have a higher nutritional status, including in their fiber content, compared to refined grain products (e.g., white flour, white bread, white pasta) [73].

To summarize, refined grain products (e.g., white flour, white bread) should be replaced by wholegrain products to increase the dietary fiber intake and promote gut and brain health.

### 4.3. Legumes

Legumes are a plant family that encompasses beans, peas, and lentils. Legumes contain protein, fiber, B vitamins, iron, folate, calcium, potassium, phosphorus, and zinc [74]. Research investigating legume consumption and their association with mental disorders and neurodegenerative diseases is relatively scarce [75]. Nevertheless, various anti-inflammatory diets promote the consumption of plant-based foods, in which legumes are an important protein source [76,77].

Legumes, rich in fiber, protein, and various micronutrients, may contribute to overall health and potentially brain health,

### 4.4. Fish

Fish are an important protein source, being high in omega-3 fatty acids. Omega-3 are essential fatty acids that are important for brain function and need to be obtained from food, particularly fatty fish species. Accumulating evidence suggests that omega-3 has an immunomodulatory capacity and protects against both depression [30,78,79,80] and dementia [81,82]. Cross-sectional analyses of the PREDIMED-Plus trial found a U-shaped relation between fatty fish consumption and the life-time prevalence of depression and the intensity of depressive symptoms [30]. These essential fats, especially eicosapentaenoic acid (EPA) and docosahexaenoic acid (DHA), are crucial for brain function and have potent anti-inflammatory effects. Omega-3 fatty acids may help stabilize mood and reduce depressive symptoms, both symptoms relevant for people with BD [83]. In SSD, omega-3 supplementation has been associated with reduced transition rates from ultra-high-risk states to psychosis [84].

A comprehensive population-based cohort study (N = 5289) found an association between unsaturated fatty acid intake, including omega-3, and a lower risk of PD [85]. A randomized double-blind placebo-controlled trial (N = 40) suggested that omega-3 fatty acids, supplied as adjuvants, slow the progression of PD [86]. Fish consumption was inversely related to dementia including AD [82,87]. A review of nine prospective studies reported an inverse association between fish consumption and AD [88]. Increasing fish intake with 100 g/week was associated with a reduction in the risk of AD by 12%. This finding is in line with another quantitative review in which fish consumption was associated with a reduced risk of dementia [87].

In summary, incorporating fish, particularly fatty fish rich in omega-3 fatty acids, into a diet is strongly associated with multiple mental and brain health benefits. The evidence highlights the role of omega-3 in reducing the risk of depression, psychosis, PD and AD. These essential fatty acids not only exhibit anti-inflammatory properties but also support cognitive function and mood stabilization.

### 4.5. Meat

Meat is a source of protein, vitamin B12, iron, and saturated fats. Meat consumption affects the microbiota and is associated with *Blautia* spp., being increased in several medical conditions including IBD [21]. Meat consumption is negatively associated with a butyrate producing-bacteria (*Roseburia* spp.), while lean beef is positively related to a SCFA-producer (*F. prausnitzii*). The consumption of processed meat, and to some extent red meat, may negatively impact CRP levels [89,90,91,92] and is associated with a small increase in the risk of depression [93].

A higher total meat consumption is associated with an increased risk of cognitive impairment, particularly with total processed meat and processed red meat. Each additional 50 g per day of total meat and processed meat intake increased the risk of cognitive impairment more [94]. Zhang et al. reviewed 29 studies on the relation between meat intake and cognitive health, finding mixed results: while most studies found no associations, some indicated reduced odds of cognitive disorders with regular meat consumption [95].

In summary, the pro-inflammatory characteristics of meat may not support mental and brain health, suggesting that the consumption of meat should be minimized.

### 4.6. Fermented Foods

Most fermented foods are considered probiotic and some are considered prebiotic as well. Fermented foods may benefit brain health [96,97]. Fermented dairy (e.g., kefir, yoghurt, buttermilk), containing live bacteria, is favored over other dairy types, since accumulating evidence has pointed to positive effects on the gut’s microbial composition and the composition of inflammatory markers [21,98]. Pre- and probiotics are likely responsible for the probable health and nutritional benefits of fermented dairy products. For example, a positive association was found between fermented dairy and SCFA-producing-bacteria [21]. Moreover, buttermilk was associated with higher microbial diversity, while the opposite was found for high-fat (whole) milk [20].

Examples of vegetable-based fermented foods include sauerkraut (fermented cabbage), kimchi (Korean fermented vegetables, typically cabbage and radishes), pickles (fermented cucumbers), olives when fermented in brine, and tempeh. Fermented foods are thought to influence brain health through multiple mechanisms, including the modulation of the gut microbiome, the production of neurotransmitters, and a reduction in inflammation [99]. These foods contain probiotics and bioactive compounds that may have neuroprotective effects [100].

In summary, the inclusion of fermented foods in the diet offers significant benefits for mental and brain health. The probiotics and bioactive compounds present in fermented foods contribute to gut health by promoting microbial diversity and reducing inflammation. These effects can support the gut–brain axis, potentially improving symptoms related to neurodegenerative diseases and mental health disorders.

### 4.7. Nuts

Nuts are high in fiber, protein, unsaturated fatty acids, various vitamins, minerals, and phytochemical compounds [101]. Nuts and seeds have shown potential benefits in mood disorders and neurodegenerative diseases [102,103,104,105,106]. In particular, walnuts are considered neuroprotective [107]. Walnuts are rich in alpha-linolenic acid (ALA), melatonin, and contain more polyphenols than other nut types. Both omega-3 fatty acids and polyphenols are important for brain health [105].

Given their nutrient density and potential neuroprotective effects, nuts—especially walnuts—may be valuable in anti-inflammatory diets for mental and neurodegenerative disorders. 

### 4.8. Olive Oil

Olive oil is the main added fat in the Mediterranean diet [108]. It is a rich source of biophenols [36,101]. Olive oil has been found to have positive effects on mental health [108,109], but its anti-inflammatory capacity in humans needs further investigation. The supplementation of an anti-inflammatory diet with extra virgin olive oil has been shown to impact global cognition and various cognitive domains [110,111,112,113].

In summar, olive oil—particularly extra virgin olive oil—shows promise in supporting mental health and cognitive function, due to its rich biophenol content and anti-inflammatory properties.

### 4.9. Salt, Herbs and Spices

A recent review investigated whether sodium intake induces systemic inflammation [114]. Different levels of sodium intake were not significantly related to changes in plasma circulating inflammatory responses including CRP, TNF-α, and IL-6. Major methodological limitations were addressed, such as high heterogeneity, the high variability in sodium level intake, and the inclusion of studies mainly conducted in non-healthy individuals [114]. The qualitative review suggested a modest association between high sodium intake and poor cognitive functioning, supported by higher quality studies.

Shivappa et al. identified several herbs and spices with anti-inflammatory potential including curcuma, ginger, garlic, thyme/oregano, rosemary and pepper [115]. In particular, curcuma and ginger have been investigated in the context of inflammation. Curcuma is known for its antioxidative, anti-inflammatory and neuroprotective properties [116]. Several meta-analyses found that curcumin or turmeric have a significant effect in reducing circulating levels of IL-6, CRP [117,118] and TNF-α [119]. Turmeric has shown promise in both depression and PD models, largely due to its active compound curcumin [120,121]. However, a more recent meta-analysis did not find that curcumin or turmeric had an effect on various inflammatory mediators in chronic inflammatory diseases [122].

Ginger may have anti-oxidative and anti-inflammatory effects [123,124], and a significant reduction in serum IL-6, CRP, TNF-α and prostaglandin E2 were observed after ginger supplementation [124]. Herbs and spices, often overlooked in dietary studies, can be potent sources of antioxidants and anti-inflammatory compounds.

To summarize, while not a primary food group, incorporating a variety of herbs and spices into the diet may provide additional anti-inflammatory benefits.

### 4.10. Beverages

Drinking water (1.5–2 L/day) helps maintain fluid balance in the body and can alleviate constipation, especially when paired with a fiber-rich diet [125].

Coffee [126,127,128,129] and green tea [115,130,131,132,133,134] contain bioactive compounds that exert anti-inflammatory, antioxidative, probiotic and neuroprotective effects.

Sugar, as in sweetened beverages, impair mental health [135,136]. Several observational studies found an association between the intake of sugar-sweetened beverages and CRP levels in a diverse group of study subjects, even after controlling for potential confounders [137,138,139,140]. A meta-analysis demonstrated that a high consumption of soft drinks is associated with an increased risk of depression [141].

Alcohol consumption, including red wine, is regarded as a double-edged sword due to its dual effect regarding (gut)inflammation and its direct toxic effects on brain cells [21,142,143].

In summary, drinking 1.5–2 L of liquids including water, coffee and tea supports brain functioning and can help against constipation. The consumption of sweetened beverages should be limited, and alcohol consumption should be avoided or consumed at a maximum of a glass per day.

### 4.11. Ultra-Processed Foods

The NOVA classification classifies foods according to their processing, rather than their nutrient content [144]. Ultra-processed foods (UPFs) are defined as “formulations of industrial sources of dietary energy and nutrients, particularly unhealthy types of fat, starches, free sugars and salt, plus additives including those designed to intensify sensory impact” [143]. These products have become a major hallmark of Western diets [145]. Examples of typical UPFs are confectionary, carbonated drinks, margarines and spreads, fruit yoghurts and drinks, reconstituted meat products, pre-prepared frozen dishes, and ‘instant’ sauces.

The primary aim of industrial processing is to produce ready-to-eat products. The consumption of UPFs had increased globally [146]. This trend is concerning because consuming these products is associated with a reduced intake of dietary fiber, phytoestrogens, and a broad range of micronutrients (e.g., vitamin A, iron, zinc) [144]. UPFs contribute to a gut ecosystem containing microbes that promote a broad range of inflammation-related diseases including metabolic and gastrointestinal disorders [144,145].

Recent research has highlighted the negative impacts of UPF on gastrointestinal health. A systematic review found that UPF consumption is associated with an increased risk of gastrointestinal disorders, including IBD [147]. A cross-sectional study reported that a higher consumption of UPFs is linked to a 26% higher risk of gastrointestinal disorders [148]. Martini et al. discussed the potential mechanisms by which UPFs might affect gastrointestinal health, including altering the composition of the gut microbiota, damaging the gut’s epithelial barrier, and increasing intestinal permeability [149]. A prospective cohort study found that for every 10% increase in UPFs in the diet, there was a 12% increase in the risk of developing IBD [150].

UPFs have been associated with increased inflammation and potentially worse outcomes in mental health disorders [14]. For example, a prospective cohort study reported that in a large cohort of French adults, the percentage of UPFs in the diet was associated with an increased risk of incident depression symptoms [151]. In addition, a meta-analysis of several observational studies showed that the consumption of UPFs was strongly associated with negative mental health, in particular with a higher risk of depression and anxiety, possibly contributing to higher rates of gastrointestinal issues in these populations [147]. In PD, while not specifically focusing on UPFs, research suggests that dietary factors can influence gut health and potentially impact disease progression [152]. Processed foods are often high in salt. Recommendations generally advocate for reduced salt intake, particularly to address hypertension, which is a risk factor for cognitive decline and dementia [153,154]. These foods often lack the beneficial nutrients found in whole foods and may contribute to gut dysbiosis and systemic inflammation.

Reducing one’s intake of UPFs may be an important strategy in dietary interventions to improve mental and brain health.

## 5. Discussion and Conclusions

This review synthesized the evidence regarding anti-inflammatory diets and their potential benefits for individuals with mental disorders and neurodegenerative diseases. Some dietary components may have beneficial effects on these disorders, primarily through neuroprotective mechanisms, an increase in the content of dietary fiber to improve gut health, and through anti-inflammatory mechanisms. Therefore, an optimal diet to improve mental and brain health should consist of food components needed by the brain for neuronal health, be rich in dietary fiber to nourish the gut microbiota and include few pro- and many anti-inflammatory products to enhance the immune system.

Key dietary components that have shown potential benefits include dietary fibers (from fruits and vegetables and whole grains), fish containing omega-3 fatty acids, fermented foods, nuts and seeds, and certain herbs and spices. These foods are generally rich in antioxidants, dietary fiber, and bioactive compounds that may help modulate inflammation, support gut health, and promote neuroprotection. Conversely, the diet recommends limiting UPFs, red meat and sugary beverages, as these are high in refined carbohydrates and/or unhealthy fats, which have been associated with gut dysbiosis, increased inflammation and potentially worse outcomes in these disorders, including gastrointestinal complaints.

The Brain Anti-Inflammatory Nutrition (BrAIN) diet is designed with these principles in mind (Table 1). In fact, the BrAIN diet may also benefit the health of people without mental disorders or neurodegenerative diseases, since it closely aligns with the Dutch guidelines for a healthy diet [155], which emphasize higher intakes of plant-based foods over animal products. The BrAIN diet adheres to these recommendations for all components, including salt and fiber intake, but differs in the following aspects: (a) it prefers fermented dairy over other dairy products; (b) it includes at least three servings of legumes per week (instead of 2–3), (c) it suggests fish twice a week (instead of 1–2 times), (d) it suggests 3–4 eggs a week (instead of 2–3), and (e) it places extra emphasis on red fruits (e.g., berries—2 times a week) and green leafy vegetables (1–2 times a week).

The Diet Inflammatory Index (DII) provides an overview of forty-five food parameters and their anti- or pro-inflammatory potential [111]. The BrAIN diet uses the DII to categorize foods based on their inflammatory score. For example, pro-inflammatory DII scores were found for saturated, trans and total fat, whereas anti-inflammatory scores were found for fiber, onion, caffeine, omega-3 fatty acids and a range of different flavonoids. Antioxidant flavonoids are one major hallmark of anti-inflammatory diets and are naturally present in plant origin foods such as vegetables, fruits, and tea [156,157]. The BrAIN diet is rich in foods high in isoflavones such as soybeans, tofu, tempeh, chickpeas and peanuts. Together with physical activity, this could provide a further alternative strategy for preventing complications and delaying the progression of noncommunicable disorders, including CVD, metabolic syndrome (highly prevalent in mental disorders) and neurodegenerative diseases [42,43].

In line with the DII, the study of Bolte et al. investigated the relations between individual foods and nutrients and the gut microbiome regarding their pro- or anti-inflammatory potential [21]. The authors of that study suggested that a specific diet can attenuate intestinal inflammation by modulating the gut microbiome: this diet comprises a higher plant than animal content and fermented dairy products, with a limited intake of processed meat and alcoholic and soft drinks.

The BrAIN diet shares many similarities with established healthy dietary patterns such as the Mediterranean diet [158,159] and the MIND diet [37] (Table 1). Our proposed dietary pattern, however, has a broader focus on mental health disorders including both psychiatric and neurodegenerative conditions, emphasizing specific components that may have particular relevance for BD, SSD and PD, such as omega-3 rich fish, anti-inflammatory herbs and spices, high amounts of dietary fibers, and fermented foods.

The potential mechanisms underlying the effects of this dietary pattern are multifaceted and interconnected. They include the modulation of the gut microbiota and the gut–brain axis, the regulation of inflammatory pathways, a reduction in oxidative stress, the influence on neurotransmitter systems, the promotion of neuroprotection and neuroplasticity, and potential epigenetic modifications. The complexity of these mechanisms underscores the potential for diet to have wide-ranging effects on brain health.

### 5.1. Limitations and Future Implications

Much of the existing research consisted of observational studies, which can establish associations but not causality. The few intervention studies we found were often small in scale and short in duration. Another limitation of any interventional diet study is its limited dietary adherence in real-world settings. While controlled studies can demonstrate the potential benefits of certain dietary patterns, translating these findings into effective interventions requires the consideration of factors such as food availability, cultural preferences, cooking skills, and socioeconomic constraints. We have designed an open-label RCT to specifically test for the beneficial effects of this diet.

### 5.2. Conclusions

In conclusion, nutritional interventions may become an important component of treatment strategies, offering a holistic approach to managing these challenging conditions.

While the evidence is still accumulating, an anti-inflammatory diet, like the BrAIN diet, rich in fruits, vegetables, whole grains, healthy fats, and fermented foods, shows promise as an adjunct in the management of mental disorders and neurodegenerative diseases. By addressing chronic inflammation, supporting gut health, and promoting neuroprotection, such a diet may help ameliorate symptoms and potentially slow disease progression. Many patients are interested in food-based strategies to cope with chronic mental disorders and neurodegenerative diseases, and clear recommendations that can easily be implemented also by patients with limited physical or budgetary resources are valuable. By incorporating a variety of anti-inflammatory foods into our diet whilst avoiding pro-inflammatory and UPFs, chronic inflammation in the body can be reduced and overall well-being promoted both for patients with mental disorders and neurodegenerative diseases, as well as for a healthy adult population. However, robust clinical trials are needed to establish the efficacy of this approach and to develop evidence-based dietary guidelines for these populations. We are currently testing the BrAIN diet in an open-label RCT in patients with BD, SSD or PD.

It will be essential to not only investigate the efficacy of anti-inflammatory diets but also to develop practical strategies for implementing dietary changes. This may involve addressing barriers to healthy eating, providing nutrition education, and considering individual preferences and cultural contexts.

## Figures and Tables

**Table 1:** Overview of BrAIN diet compared to other anti-inflammatory diets on the food group level, with high (green), moderate (yellow) or limited (orange) recommendation.

Amounts	MedDiet	MIND	BrAIN	
High	Vegetables	Green leafy vegetables Other vegetables	Vegetables Incl. green leafy and cruciferous vegetables, onion, garlic	**Fiber-rich foods**
	Fruit -	- Berries	Fruit Incl. berries	
	Legumes	Beans	Legumes	
	Nuts and seeds -	Nuts -	Nuts Incl. walnuts	
	Wholegrains	Wholegrains	Wholegrains	
	Fish	Fish	Fish	
	-	-	Fermented dairy	
	-	Poultry	-	
	Olive oil	Olive oil	Olive oil	
	- -	- -	Herbs and spices Incl. curcuma, ginger, pepper, thyme, oregano, rosemary	
Moderate	Dairy products	-	-	
	Poultry	-	Lean meat/poultry	
	Alcohol/red wine	Alcohol/wine	-	
	-	-	Coffee	
Limited	Red meat	Red meat (products)	Red meat	
	Processed meat	-	Processed meat	
	(Ultra-)processed foods Sweets	- Sweets, fried foods	(Ultra-)processed foods Incl. sugar sweetened beverages	
	-	Cheese, butter/margarine	-	

MedDiet = Mediterranean diet; MIND = Mediterranean–DASH Diet Intervention for Neurodegenerative Delay; BrAIN = Brain Anti-Inflammatory Nutrition.

## Data Availability

Data sharing not applicable to this article as no datasets were generated or analyzed during the current study.
